# Supplementary material for: Impact of Metacognitive and Psychological Factors in Learning-Induced Plasticity of Resting State Networks
Source: Biology (Basel). 2022 Jun 10;11(6):896. doi: 10.3390/biology11060896 (PMC9219664; doi:10.3390/biology11060896)
Supplement: Supplementary file 1 [file biology-11-00896-s001.zip › biology-1730661-supplementary.pdf]

Supplementary materials

Supplementary Table S1: Factor's loading of the psychological variables (loading < 0.3 are left blanked).

	Factor	
	1: « Anxiety sensitivity »	2: « Achievement sensitivity »
STAI_T	**0.879**	0.651
SPSRQ_P	**0.851**	
STAI_S	**0.795**	
HAD_A	**0.743**	0.465
FMPS_PE	0.585	**0.866**
FMPS_SP		**0.723**
SPSRQ_R		**0.651**
HAD_D	0.551	**0.660**
FMPS_DA	0.494	**0.632**

Supplementary Table S2: Simple correlations (r) between the Δ FC in the "confidence network", age and education.

	R Crus2 - L Frontal SupOrb	R Angular – R Frontal Inf. Oper.	R Hippocampus – R Cereb. 9	R Amygdala – R Cereb. 9	R Amygdala – L Cereb. 9	R Amygdala - R Angular	R Amygdala - R Precuneus	R Amygdala - L Angular	L Putamen - R Angular	L Cereb. 9 – R Cereb. 9	R Cereb. 6 – R Cereb.9
Age	-0.473 **	-0.363 *	-0.240	-0.426*	-0.329	-0.193	-0.369*	-0.184	-0.166	-0.384*	-0.323
Education	0.244	0.156	0.428*	0.363	0.473*	0.245	0.374	0.179	0.342	0.076	0.302

Note: * p <0.05; ** p < 0.01; *** p < 0.001

Supplementary Table S3: Partial correlations (r) between the Δ FC in the "confidence network" and "Anxiety Sensitivity" factorial scores while controlling for age and education.

	R Crus2 – L Frontal SupOrb	R Angular – R Frontal Inf. Oper.	R Hippocampus – R Cereb. 9	R Amygdala – R Cereb. 9	R Amygdala – L Cereb. 9	R Amygdala - R Angular	R Amygdala - R Precuneus	R Amygdala - L Angular	L Putamen - R Angular	L Cereb. 9 – R Cereb. 9	R Cereb. 6 – R Cereb. 9
Factor 1	-0.389	-0.463*	-0.371	-0.390	-0.551 **	-0.466 *	-0.316	-0.517 *	-0.486 *	-0.485*	-0.499*

Note: * p <0.05; ** p < 0.01; *** p < 0.001

Supplementary Table S4: Partial correlations (r) between the Δ FC in the "confidence network" and "Anxiety Sensitivity" factorial scores while controlling for learning accuracy, age and education.

	R Crus2 - L Frontal SupOrb	R Angular – R Frontal Inf. Oper.	R Hippocampus – R Cereb. 9	R Amygdala – R Cereb. 9	R Amygdala – L Cereb. 9	R Amygdala - R Angular	R Amygdala - R Precuneus	R Amygdala - L Angular	L Putamen - R Angular	L Cereb. 9 – R Cereb. 9	R Cereb. 6 – R Cereb. 9
Factor 1	- 0.339	-0.434*	-0.303	-0.329	-0.523 *	-0.404	-0.257	-0.485 *	-0.447 *	-0.471 *	-0.453*

Note: * p <0.05; ** p < 0.01; *** p < 0.001

Supplementary Table S5: Partial correlations (r) between the functional connectivity strength at rest in the "confidence networks" and "Anxiety Sensitivity" factorial scores while controlling for age and education.

	R Crus2 - L Frontal SupOrb	R Angular – R Frontal Inf. Oper.	R Hippocampus – R Cereb. 9	R Amygdala – R Cereb. 9	R Amygdala – L Cereb. 9	R Amygdala - R Angular	R Amygdala - R Precuneus	R Amygdala - L Angular	L Putamen - R Angular	L Cereb. 9 – R Cereb. 9	R Cereb. 6 – R Cereb.9
Factor 1	-0.021	0.163	0.155	0.353	0.335	0.474*	0.329	0.372	-0.038	0.256	0.449*

Note: * p <0.05; ** p < 0.01; *** p < 0.001

Supplementary Figure S1: sagittal, coronal and axial views of the "learning accuracy network"

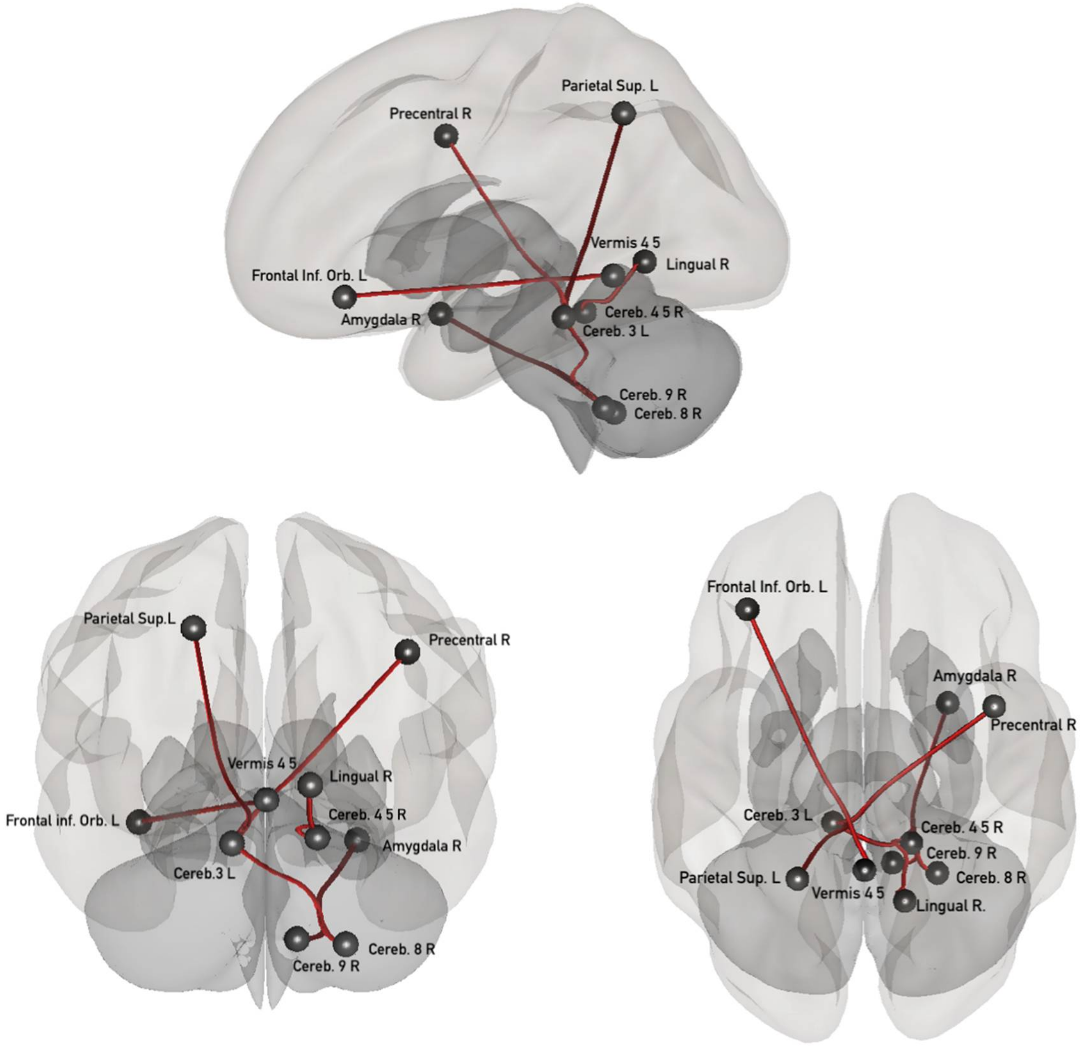

L: left; R: right; Inf: inferior; Sup: superior; Cereb.: Cerebellum; Orb: Orbital; Oper.:Opercular.
